# Formation of contact and multiple cyclic cassiterite twins in SnO_2_-based ceramics co-doped with cobalt and niobium oxides

**DOI:** 10.1107/S2052520622006758

**Published:** 2022-07-27

**Authors:** Nina Daneu, Goran Dražič, Matjaž Mazaj, Fabrice Barou, José Alberto Padrón-Navarta

**Affiliations:** a Advanced Materials Department, Jožef Stefan Institute, Jamova cesta 39, Ljubljana, 1290, Slovenia; b Materials Chemistry, National Institute of Chemistry, Hajdrihova 19, Ljubljana, Slovenia; c Inorganic Chemistry and Technology, National Institute of Chemistry, Hajdrihova 19, Ljubljana, Slovenia; d Géosciences Montpellier, Université de Montpellier and CNRS, UMR5243, Montpellier, France; e Andalusian Institute of Earth Sciences, Spanish Research Council and University of Granada, Granada, Spain; Moscow State University, Russian Federation

**Keywords:** growth twinning, contact twins, cyclic twins, nucleation twinning, orientation relationship

## Abstract

Contact and multiple cyclic twins of cassiterite commonly form in SnO_2_-based ceramics when SnO_2_ is sintered with small additions of cobalt and niobium oxides (dual doping). In this work, it is shown that the formation of twins is a two-stage process that starts with epitaxial growth of SnO_2_ on CoNb_2_O_6_ and Co_4_Nb_2_O_9_ seeds (twin nucleation stage) and continues with the fast growth of (101) twin contacts (twin growth stage). Both secondary phases form below the temperature of enhanced densification and SnO_2_ grain growth; CoNb_2_O_6_ forms at ∼700°C and Co_4_Nb_2_O_9_ at ∼900°C. They are structurally related to the rutile-type cassiterite and can thus trigger oriented (epitaxial) growth (local recrystallization) of SnO_2_ domains in different orientations on a single seed particle. While oriented growth of cassiterite on columbite-type CoNb_2_O_6_ grains can only result in the formation of contact twins, the Co_4_Nb_2_O_9_ grains with a structure comparable with that of corundum represent suitable sites for the nucleation of contact and multiple cyclic twins with coplanar or alternating morphology. The twin nucleation stage is followed by fast densification accompanied by significant SnO_2_ grain growth above 1300°C. The twin nuclei coarsen to large twinned grains as a result of the preferential and fast growth of the low-energy (101) twin contacts. The solid-state diffusion processes during densification and SnO_2_ grain growth are controlled by the formation of point defects and result in the dissolution of the twin nuclei and the incorporation of Nb^5+^ and Co^2+^ ions into the SnO_2_ matrix in the form of a solid solution. In this process, the twin nuclei are erased and their role in the formation of twins is shown only by irregular segregation of Co and Nb to the twin boundaries and inside the cassiterite grains, and Co,Nb-enrichment in the cyclic twin cores.

## Abstract 2

The formation of contact and cyclic cassiterite twins during crystal growth is controlled by oriented nucleation of SnO_2_ domains on nanosized grains of structurally related cobalt–niobium oxide phases.

## 1. Introduction

Understanding the formation of twins during crystal growth, *i.e.* growth twins is one of the fundamental challenges in mineralogy and materials science that remains incompletely understood (Shahani & Voorhees, 2016 ▸). The presence of growth twins influences the crystal morphology via the development of reentrant angles and, it is commonly observed that growth twins are larger than untwinned crystals due to their faster growth along the twin boundary planes (Shahani & Voorhees, 2016 ▸; Otálora & García-Ruiz, 2014 ▸). Both effects suggest that growth twins have the potential to be exploited for the fabrication of materials with tailored properties via controlling the crystal shape and size. Therefore, it is important to understand the process of growth twinning in detail.

**Figure 1:**
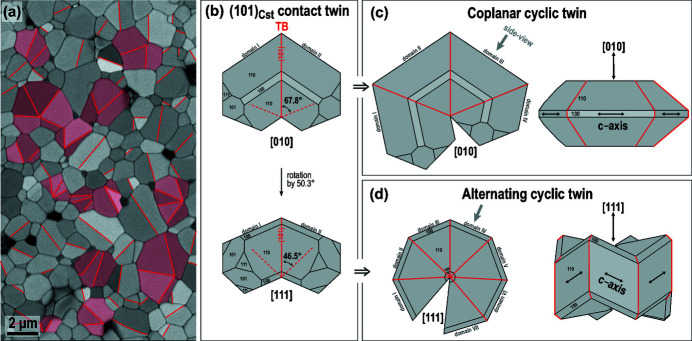
(*a*) Typical microstructure of cobalt and niobium oxide-doped SnO_2_-based ceramics composed of cassiterite (SnO_2_) grains that frequently contain (101) twin boundaries (TBs; marked by red lines). Most grains are untwinned, some contain a single TB, these are contact (101) twins, and some grains contain two or more TBs, these are multiple cyclic twins (shaded in red). (*b*) A (101)_Cst_ contact twin viewed along the [010]_Cst_ and [111]_Cst_ common zone axis. Dashed red lines indicate edge-on oriented {101}_Cst_ reflection planes in the twin domains. (*c*) Coplanar cyclic twin of cassiterite with four domains viewed along the common [010]_Cst_ axis. The side-view reveals that the tetragonal *c* axes of all domains lie in the same plane. (*d*) Alternating cyclic twins of cassiterite contain up to seven domains (see Padrón-Navarta *et al.* (2020 ▸) for detailed description) oriented along their [111]_Cst_ zone axes. In this type of cyclic twins, the *c* axes of twin domains alternate up and down as visible in the side view of the twin.

This work addresses the origin of contact and multiple cyclic twins of cassiterite (SnO_2_) that form in polycrystalline SnO_2_-based ceramics when SnO_2_ is sintered with small amounts of cobalt oxide and niobium oxide (dual-doped SnO_2_). The typical microstructure of SnO_2_ with the addition of 1 mol% of CoO and 1 mol% of Nb_2_O_5_ after sintering at 1430°C for 5 h is shown in Fig. 1 ▸(*a*). In our previous studies, we described microstructure development and electrical properties of the ceramics (Tominc *et al.*, 2018 ▸), and analyzed the morphology of multiple twins using electron back-scatter diffraction (EBSD) (Padrón-Navarta *et al.*, 2020 ▸). We have shown that small addition of both aliovalent dopants in the Co:Nb ratio of up to 1:2 is necessary for efficient densification of the ceramics and growth of SnO_2_ grains (Tominc *et al.*, 2018 ▸). We also observed that dual doping results in the abundant formation of (101) twin boundaries (TBs) in cassiterite grains during SnO_2_ grain growth (Tominc *et al.*, 2018 ▸; Padrón-Navarta *et al.*, 2020 ▸). Grains with a single (101) TB or contact twins are the most common among the twinned grains, whereas multiple cyclic twins represent only around 6% of all grains. Cyclic twins of two types were identified by EBSD, coplanar and alternating, both types occur in similar fractions and are randomly distributed in the microstructure.

Multiple twins are frequently observed in rutile (TiO_2_) and cassiterite. The minerals are isostructural and are characterized by the 4/*m* 2/*m* 2/*m* point group, which has four equivalent {101} reflection planes and each can represent a twin contact. This may lead to the formation of subsequent multiple twins with a variety of morphologies (see chapter 3.3.6.9. in Hahn & Klapper, 2006 ▸). In this work, we focus on a special type of multiple twins, *i.e*. the cyclic twins, in which all TBs radiate from a common twin core. Both types of cyclic twins, with coplanar and alternating morphology, can be derived from a contact (101) twin as schematically shown in Figs. 1 ▸(*b*)–1 ▸(*d*). Fig. 1 ▸(*b*) is a (101)_Cst_ contact twin oriented along the [010]_Cst_ and [111]_Cst_ zone axes common to both twin domains. In every orientation, two sets of {101}_Cst_ reflection planes are edge-on oriented (in each twin domain), and the other {101}_Cst_ reflection planes are oblique to the viewing direction. Cyclic twins form when twin contacts develop on subsequent edge-on oriented {101} reflection planes with all domains aligned along the same zone axis. In coplanar cyclic twins, the twin domains have the [010]_Cst_ axis in common [Fig. 1 ▸(*c*)], whereas, in alternating cyclic twins, all twin domains are oriented along their [111]_Cst_ zone axis [Fig. 1 ▸(*d*)]. A more detailed description of both types of multiple cyclic twins of cassiterite that occur in the dual-doped SnO_2_ is given by Padrón-Navarta *et al.* (2020 ▸).

Twins of different morphology and origin have been described in synthetic SnO_2_. Deformation twins on {101} planes can be introduced into SnO_2_ (and also in isostructural rutile, TiO_2_) crystals by mechanical grinding (Suzuki *et al.*, 1991 ▸). Twinning during crystal growth was also reported; for example, contact, polysynthetic, and cyclic growth twins on {101} planes were found in nanocrystalline SnO_2_ thin films produced from amorphous oxygen-deficient SnO_2_ indicating the role of nonstoichiometry in the twinning process (Zheng *et al.*, 1996 ▸). Abundant {101} twins in epitaxial thin films of SnO_2_ produced from α-SnO precursor on 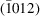 sapphire (Al_2_O_3_) substrate formed as a result of oriented growth and recrystallization of α-SnO to SnO_2_ (Pan & Fu, 2001 ▸). Contact twins also formed in Cu_2_O flux-grown SnO_2_ crystals with the addition of trivalent (Fe^3+^) and pentavalent (Nb^5+^, Ta^5+^) cations suggesting the role of these dopants in the formation of the twins (Kawamura *et al.*, 1999 ▸). In our previous work, we analyzed (101) TBs in the dual-doped SnO_2_ (Tominc *et al.*, 2018 ▸) by aberration-corrected scanning transmission electron microscopy and confirmed that the concentration of Co and Nb along the TB planes is slightly increased but the distribution of both elements within the interface is not uniform (Tominc *et al.*, 2018 ▸). This indicates that these twins are not chemically induced (or impurity-induced) planar defects like, for example, basal-plane inversion boundaries in Sb-doped ZnO (Rečnik *et al.*, 2001*a* ▸) or {111} twins in Be-doped MgAl_2_O_4_ spinel (Drev *et al.*, 2013 ▸). The fact that all TBs in cyclic twins radiate from a common center suggests their formation by nucleation at the beginning of crystal growth; however, the reasons for the formation of different types of cyclic twins were not revealed yet.

Twinning has been even more thoroughly investigated in isostructural rutile, TiO_2_ (Rt). Here, the effect of polymorphism on the formation of twins has been identified in natural as well as in synthesized samples. Nano-scale lamellae of the high-pressure and high-temperature polymorph of TiO_2_ with the α-PbO_2_ structure were found at {101} TBs in rutile grains in ultrahigh-pressure metamorphic rocks (Hwang *et al.*, 2000 ▸; Meng *et al.*, 2008 ▸)^,^. Penn & Banfield (1998 ▸) have shown that oriented attachment of polymorphic TiO_2_ nanoparticles can result in twinning and oriented intergrowths. Another mechanism leading to rutile crystals/domains in twinned orientation is oriented (topotaxial) recrystallization and/or epitaxial growth of/on structurally related precursor minerals (Armbruster, 1981 ▸; Force *et al.*, 1996 ▸). Oriented recrystallization of ilmenite (Ilm) FeTiO_3_ with [101]_Rt_ or [001]_Rt_ parallel to [210]_Ilm_ results in the formation of complex reticulated (sagenite) rutile networks composed of twin contacts on {101} and {301} planes (crystallographic contacts) or rutile domains intersecting at 60 or 120° (non-crystallographic contacts) (Rečnik *et al.*, 2015 ▸; Stanković *et al.*, 2016 ▸). In principle, oriented recrystallization can result in the development of coplanar cyclic twins of rutile, which can be theoretically composed of up to six rutile domains, separated by five {101} TBs and an additional non-crystallographic contact (Hahn & Klapper, 2006 ▸; Padrón-Navarta *et al.*, 2020 ▸). Besides coplanar cyclic twins, alternating twins with non-planar fourfold (tetragonal) *c* axes and composed of eight domains due to the 45° angle between the (101) and 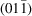 planes (Hahn & Klapper, 2006 ▸) are also found in rutile. Well known examples of these rare natural occurrences of symmetrically developed eightlings from Magnet Cove (Arkansas, USA). According to Hahn & Klapper (2006 ▸), Magnet Cove eightlings are nucleation twins, which originate from a common point (nucleus); however, the nature of the nucleus has not been described.

While the formation of certain types of twins in rutile-type minerals is well understood, the origin of cyclic twins is still puzzling the researchers. In this work, we aim to understand the formation mechanism of different types of twins that form in SnO_2_ when it is doped with cobalt and niobium oxides (Tominc *et al.*, 2018 ▸). Twins, especially the multiple cyclic twins, abundantly form only in the dual-doped SnO_2_ and their morphology is typical of nucleation twinning. To confirm the origin of cyclic twins by nucleation, we first analyzed the core of a cyclic twin by scanning transmission electron microscopy (STEM). Then we studied phase relations in SnO_2_ with higher additions of cobalt and niobium oxides to reveal which secondary phases that could potentially trigger the formation of cyclic twins form during the sintering. Based on the results we explain the formation mechanism of contact and cyclic twins by epitaxial recrystallization of SnO_2_ on particles of two secondary phases that form during the sintering, CoNb_2_O_6_ and Co_4_Nb_2_O_9_, which are both structurally related to cassiterite and describe the development of microstructures composed of normal (untwinned) grains and twins.

## 2. Experimental

### 2.1. Synthesis of samples

For our studies of core regions of cyclic twins, we used SnO_2_ doped with low amounts of cobalt oxide and niobium oxide, where the formation of large and abundantly twinned cassiterite grains is observed [see microstructure in Fig. 1 ▸(*a*), and also Tominc *et al.* (2018) ▸ and Padrón-Navarta *et al.* (2020 ▸)]. The samples were prepared by the standard solid-state synthesis, where SnO_2_ powder (Alfa Aesar, 99.9%, nanopowder) with the addition of 1 mol% CoO (Alfa Aesar, 95%) and 1 mol% Nb_2_O_5_ (Merck, 99%) was homogenized in absolute ethanol, dried and pressed into pellets. The pellets were sintered at 1430°C for 5 h in air.

For the studies of phase changes within the SnO_2_–Co_3_O_4_–Nb_2_O_5_ system and to obtain a deeper insight into the role of secondary phases on the formation of twins in dual-doped cassiterite, we prepared compositions with higher additions of both oxides. Two series of samples with Co:Nb ratio for the formation of CoNb_2_O_6_ and Co_4_Nb_2_O_9_ phases (Co:Nb ratio 1:2 and 2:1, respectively) were prepared. In both series we added 10, 25 and 50 mol% of Co_3_O_4_ (Alfa Aesar, 99.7%) and Nb_2_O_5_ mixture, in the ratio for the formation of the targeted cobalt–niobium oxide phase, to the SnO_2_ powder. The initial compositions of all samples are given in Table 1 ▸. In preparation of these compositions, we considered only the Co:Nb ratio and disregarded the oxygen nonstoichiometry due to the addition of Co_3_O_4_ instead of CoO, since Co^3+^ in Co_3_O_4_ is reduced to Co^2+^ above 800°C (Navrotsky *et al.*, 2010 ▸). The powder mixtures were homogenized in absolute ethanol, pressed into pellets, and fired at 700, 800, 900, 1000, 1100, 1200 and 1300°C for 16 h in air. After each firing, the samples were crushed, homogenized in absolute ethanol, dried, and re-pressed into pellets for firing at higher temperature.

### 2.2. Characterization techniques

Atomic scale analyses of the low-doped cassiterite ceramics were performed using aberration-corrected scanning transmission electron microscopy (STEM; ARM 200CF, Jeol Ltd, Tokyo, Japan) equipped with a high-angle annular dark-field detector (HAADF) for Z-contrast imaging and energy-dispersive X-ray spectrometer (EDXS; Jeol Centurio) with 100 mm^2^ silicon drift detector for chemical analysis at the nanoscale. Samples for STEM analyses were prepared by thinning, dimpling, and Ar ion-milling to perforation (Gatan PIPS 691, California, USA).

X-ray powder diffraction (XRD) was used for the determination of the phase composition of the samples. The analyses were performed on Bruker AXS, D4 Endeavor diffractometer, within an angular range of 2Θ from 10° to 70° with a step size of 0.04° and collection time of three seconds per step. The obtained data were used for the quantification of phases by Rietveld refinement using *Topas Academic v.6* software package. The quantification refinement included the whole pattern profile fit in the first step followed by unit-cell parameters fit. Additionally, temperature parameters and cation positions were fitted for the involved structural models due to the certain degree of cation disorder expected in the sintered phases.

Densification of the samples was monitored by a hot-stage microscope (EM201x, Hesse instruments). The diameter of a cylindrical sample with initial dimensions of 8 mm diameter and 5 mm height was measured during heating with 5 K min^−1^ up to 1450°C.

EBSD analyses were performed on CamScan X500FE Crystal Probe with inclined column optimized for EBSD and field-emission electron gun allowing high-resolution spatial analyses. The EBSD patterns were recorded at 20 kV accelerating voltage and a working distance of 20 mm. The patterns were indexed automatically using the *AZtechHKL* software package from Oxford Instruments. See Padrón Navarta *et al.* (2020 ▸) for more details about the EBSD analyses.

## 3. Results and discussion

### 3.1. STEM/EDXS analysis of the cyclic twin core

**Figure 2:**
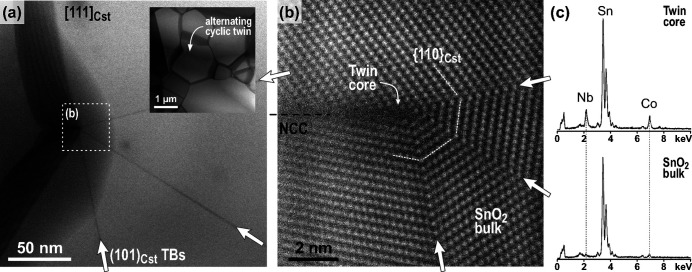
STEM/EDXS analysis of an alternating cyclic twin with four domains. (*a*) Low-magnification STEM image of the twinned grain viewed along the common [111]_Cst_ zone axis. The three (101)_Cst_ TBs and the NCC extend from a common point located near the edge of the grain. (*b*) HAADF-STEM image of the region around the core. (*c*) EDXS point analyses from the cyclic twin core and bulk SnO_2_ indicate significant enrichment with Nb and Co in the core region.

The most typical morphological feature of cyclic twins of cassiterite that form in the dual-doped SnO_2_-based ceramics is the common origin (nucleation point) of all TBs in cyclically twinned grains [see Fig. 1 ▸(*a*) and Padrón Navarta *et al.* (2020 ▸). This suggests that all TBs form on a common nucleus at the beginning of crystal growth. To find the nucleus in the twin core of cyclic twins, we analyzed the low-doped ceramics by atomic resolution STEM. The task is challenging because cyclic twins represent only about six percent of all grains (Padrón-Navarta *et al.*, 2020 ▸) and hence the probability of finding a cyclic twin, which is oriented close to the common zone axis ([010] for coplanar and [111] for alternating twins), with a visible nucleation core and, at the same time, located in the thin part of the sample, is relatively low. An example of an alternating cyclic twin with four domains, located in a slightly thicker, but still electron-transparent part of the sample and oriented along the common [111] zone axis is shown in Fig. 2 ▸.

The cyclic twin contains three TBs extending from a common center, which is positioned near the edge of the grain [Fig. 2 ▸(*a*)]. Such eccentric position of the twin core is typical for cyclic twins in cassiterite ceramics as previously observed based on SEM and EBSD analyses (Tominc *et al.*, 2018 ▸; Padrón-Navarta *et al.*, 2020 ▸). A high-resolution HAADF-STEM image taken around the twin core region is shown in Fig. 2 ▸(*b*). The bright contrast stems mainly from the heavy Sn atomic columns (cation substructure) and shows that all three TBs are edge-on oriented and lie in subsequent edge-on oriented (101) planes with a 46.5° angle between two planes. At this magnification, the common origin point of all TBs of the cyclic twin is even more obvious. All three TBs are mirror-symmetric across the twin plane. This atomic configuration of the TB corresponds to the lowest-energy pseudo-twin model of {101} twins characterized by in-plane translation of ½〈111〉 in SnO_2_ (Lee *et al.*, 1993 ▸). In addition to the three twin boundaries (crystallographic contacts), the cyclic twin contains an additional non-crystallographic contact (NCC) between the first and the last (fourth) domain, which is incomparably shorter than the twin boundaries [Figs. 2 ▸(*a*) and 2 ▸(*b*)]. This is due to the lower degree of local atomic ordering at the NCC in comparison to crystallographic contacts like twin boundaries and hence such an interface is not energetically favored. The core region contains no visible secondary crystalline phase and can be described as a nanosized amorphous pocket [Fig. 2 ▸(*b*)]. EDXS spectra were recorded in bulk SnO_2_ and in the core [Fig. 2 ▸(*c*)]. While bulk SnO_2_ contains only low amounts of Co and Nb in the form of solid solubility, the amorphous core region is significantly enriched in Co and Nb. Quantification of EDXS spectra was unreliable due to the relatively large thickness at the area of interest, low count ratio, and inconvenient sample tilt relative to the detector. EDXS analysis was performed also on the TBs, where segregation of Co or Nb was not detected by EDXS, however previous analyses of this sample using quantitative HAADF-STEM indicated low and nonuniform segregation of Co and Nb to the TB planes (Tominc *et al.*, 2018 ▸).

Excess of Co and Nb detected in the core of the cyclic twin, however, does suggest the presence of a Co- and Nb-rich precursor phase during the nucleation stage. The question is why there is no crystalline Co–Nb phase in the core. One of the possible explanations is that the cross-section did not exactly intersect the nucleus, which has been located above or below the thin section. Another possibility is that a seed particle existed before the growth of the cyclic twin when it served as a twin nucleation core, and later, in the process of SnO_2_ crystal growth at higher temperatures, it reacted with SnO_2_ causing the incorporation of Co and Nb in the bulk cassiterite in the form of solid solution. This process is even more likely in the samples with low doping rates, where the addition of both dopants does not exceed the solid solubility limits of Co and Nb in SnO_2_ (the estimated value of total Co and Nb solid solubility in SnO_2_ is around 4–6 at%) and therefore most of the Co and Nb is incorporated into SnO_2_ in the ∼1:2 to 1:4 ratio. The total amount of Co and Nb and the Co:Nb ratio vary within these limits inside the SnO_2_ grains, whereas excess cobalt oxide reacts with SnO_2_ to Co_2_SnO_4_ spinel grains (Tominc *et al.*, 2018 ▸).

The fact that cyclic twins are observed only in the samples with the addition of both aliovalent oxides (cobalt and niobium oxide) is a strong indication that the nucleation of cyclic twins is related to the formation of a cobalt–niobium oxide. The formation of such a secondary phase in the form of nanosized nuclei and its transient stability is another possible scenario of twin formation, and would agree with the theory about the formation of cyclic twins by nucleation proposed by Senechal (1980 ▸). To check the hypothesis we prepared SnO_2_ samples with higher additions of Co_3_O_4_ and Nb_2_O_5_ and investigated which phases form during the heating and how they relate to twinning in cassiterite.

### 3.2. Phases in SnO_2_ with higher additions of Co_3_O_4_ and Nb_2_O_5_

**Figure 3:**
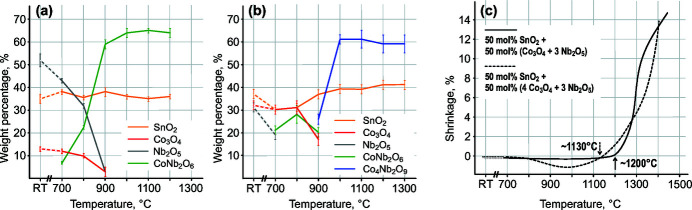
Phase changes during firing of SnO_2_ with the addition of 50 mol% of Co_3_O_4_ and Nb_2_O_5_ in the ratio for the formation of (*a*) CoNb_2_O_6_ phase and (*b*) Co_4_Nb_2_O_9_ phase. (*c*) Densification curves of both compositions.

According to the literature, CoNb_2_O_6_, Co_3_Nb_4_O_14,_ and Co_4_Nb_2_O_9_ are cobalt–niobium oxides that form during the sintering of CoO–Nb_2_O_5_ in air (Goldschmidt, 1960 ▸; Weitzel, 1976 ▸; Castellanos *et al.*, 2006 ▸). CoNb_2_O_6_ crystallizes in the columbite-type structure (abbreviated as Col-t^1^), the structure of Co_3_Nb_4_O_14_ is comparable to that of rutile, and the structure of Co_4_Nb_2_O_9_ is comparable to that of corundum (we use abbreviation Crn-t for this phase). CoNb_2_O_6_ and Co_4_Nb_2_O_9_ are both interesting in connection with nucleation twinning in cassiterite. We first synthesized both phases from stoichiometric amounts of initial oxides (Co_3_O_4_ and Nb_2_O_5_, taking into account the Co:Nb atomic ratio) at 1200°C (results not shown here). To follow phase changes in the SnO_2_–Co_3_O_4_–Nb_2_O_5_ system with increasing temperature, we studied compositions that contained 10, 25 and 50 mol% of Co_3_O_4_ and Nb_2_O_5_ in the ratio for the formation of CoNb_2_O_6_ and Co_4_Nb_2_O_9_ (Co:Nb ratios 1:2 and 2:1, see Table 1 ▸). Here we show only the results of XRD analyses for the samples with 50 mol% of both dopant mixtures because in these samples the phase changes are best visible due to the high addition of both oxides. The XRD patterns are shown in Fig. S1. XRD patterns of the samples with 10 and 25 mol% are similar, only the amount of secondary phases is lower. The phase composition of the samples with 50 mol% addition of the dopants mixture after firing at each temperature was quantified using the Rietveld refinement procedure and the results are shown in Figs. 3 ▸(*a*) and 3 ▸(*b*).

In the sample with the addition of cobalt and niobium oxides with Co:Nb ratio 1:2 [Fig. 3 ▸(*a*)], the CoNb_2_O_6_ phase starts to form already below 700°C. The reaction is completed at 1000°C, when reflections from the starting oxides Co_3_O_4_ and Nb_2_O_5_ are not observed anymore and only reflections from rutile-type SnO_2_ and CoNb_2_O_6_ are present. The two phases coexist up to 1200°C. After sintering at 1300°C, the reflections from both phases almost completely disappear (a small amount of SnO_2_ remains unreacted) and a new phase is formed. Reflections of this phase could not be matched with any phase in the available databases. Preliminary analyses with SEM/EDXS have shown that the phase has an approximate composition of SnCoNb_2_O_8_ and according to the XRD pattern, it has a similar structure to the low-temperature form of FeNb_2_O_6_, which has the trirutile structure (Aruga *et al.*, 1985 ▸; Beck, 2012 ▸). This suggests that the columbite-type CoNb_2_O_6_ phase reacts with SnO_2_ above 1200°C to form a structurally similar quaternary oxide.

In the second series of compositions, the Co_3_O_4_ and Nb_2_O_5_ were added to SnO_2_ in the ratio for the formation of Co_4_Nb_2_O_9_ phase (Co:Nb = 2:1). The results of Rietveld analysis of XRD patterns are shown in Fig. 3 ▸(*b*). Also here, the columbite-type CoNb_2_O_6_ is the first secondary phase that forms in the system already below 700°C. The fraction of this phase increases at 800°C; however, at 900°C its amount starts to decrease and reflections of the Co_4_Nb_2_O_9_ phase (Bertaut *et al.*, 1961 ▸) appear as a result of the reaction between CoNb_2_O_6_ and the remaining cobalt oxide. After sintering at 1000°C, only SnO_2_ and Co_4_Nb_2_O_9_ are present in the sample and their amount is stable up to 1300°C (after sintering at 1400°C, the sample with the highest addition of Co and Nb-oxide melted). SEM/EDXS analysis has shown around 3–4 at% Sn in Co_4_Nb_2_O_9_ and 4–6 at% of Co and Nb in 1:2 ratio in cassiterite grains in all samples, therefore these values are the approximate (estimated) solid solubility limits.

In ceramics, grain growth is closely related to diffusion processes and accelerated shrinkage. Densification characteristics of both compositions are shown in Fig. 3 ▸(*c*) and indicate that both compositions start to densify above 1100°C. In SnO_2_-based ceramics with lower additions of both dopants, the onset of densification is shifted even to temperatures above 1300°C (Tominc *et al.*, 2018 ▸). These temperatures are much higher than the formation of cobalt niobium oxides and indicate that SnO_2_ grain growth occurs much after the formation of both secondary phases.

**Figure 4:**
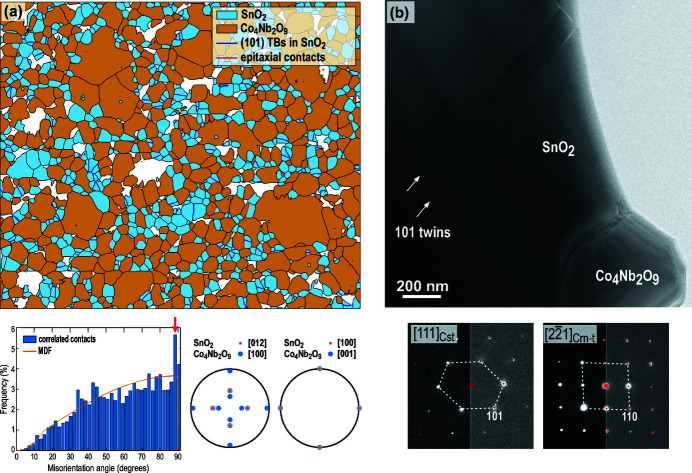
(*a*) Phase-colored EBSD map of SnO_2_ with 50% addition of Co_3_O_4_ and Nb_2_O_5_ in the ratio for the formation of Co_4_Nb_2_O_9_ phase sintered at 1300°C. Blue lines are (101) TBs in SnO_2_ grains, whereas red lines indicate epitaxial contacts between SnO_2_ (blue) and Co_4_Nb_2_O_9_ (brown) with (101)_Cst_ || (110)_Crn-t_ and (010)_Cst_ || (001)_Crn-t_. The frequency of these contacts is above the uniform misorientation distribution function (MDF) as shown in the histogram below the map. (*b*) TEM image of the sample with the 10% addition of both oxides in the same ratio shows a SnO_2_ grain in contact with Co_4_Nb_2_O_9_ particle. Selected area diffraction patterns recorded from both phases disclose epitaxial OR between both phases: 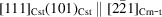 (110)_Crn-t_. This OR is identical to the epitaxial contacts determined by EBSD.

The sample with 50% addition of Co_3_O_4_ and Nb_2_O_5_ in the ratio for the formation of Co_4_Nb_2_O_9_ phase after sintering at 1300°C was analyzed by EBSD, and the sample with 10% addition of both dopants in the same ratio was inspected by TEM. Fig. 4 ▸(*a*) is EBSD phase map of the sample showing grains of the two identified phases, SnO_2_ and Co_4_Nb_2_O_9_ in blue and brown, respectively. The grain misorientation analysis has confirmed the presence of (101) twin boundaries in cassiterite grains. Interestingly, cyclic twins, especially those with more than three subsequent domains are quite rare in this sample in comparison to the low-doped ceramics (Tominc *et al.*, 2018 ▸; Padrón-Navarta *et al.*, 2020 ▸). This suggests that the conditions for the formation of cyclic twins are more easily fulfilled in samples with lower dopant additions. EBSD data was further used for the analysis of the orientation relationship (OR) between cassiterite and Co_4_Nb_2_O_9_ grains. The misorientation angle analysis of the SnO_2_–Co_4_Nb_2_O_9_ contacts [Fig. 4 ▸(*a*)] reveals that certain orientations between the two phases occur with the probability that is above the uniform misorientation distribution function (MDF) indicating preferential formation of correlated orientation relationship between grains of these two phases. The analysis of the misorientation data using MTEX toolbox (Hielscher & Schaeben, 2008 ▸; Mainprice *et al.*, 2015 ▸; Krakow *et al.*, 2017 ▸; Grimmer, 1979 ▸; Morawiec, 1997 ▸) has shown that these misorientations are crystallographic contacts that follow the OR: (101)_Cst_ || (110)_Crn-t_ and (010)_Cst_ || (001)_Crn-t_. In Fig. 4 ▸(*a*), these contacts are marked by red lines. Epitaxial OR between SnO_2_ and Co_4_Nb_2_O_9_ was also found in TEM. Fig. 4 ▸(*b*) shows a contact between a larger SnO_2_ grain and a smaller Co_4_Nb_2_O_9_ particle. The cassiterite grain is oriented along the [111]_Cst_ zone axis, while the Co_4_Nb_2_O_9_ grain along the pseudocubic 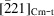 zone axis with (101)_Cst_ planes parallel to the (110)_Crn-t_ planes of Co_4_Nb_2_O_9_. This OR is equivalent to the one determined by EBSD and confirms that cassiterite grains preferentially from this type of epitaxial contact with Co_4_Nb_2_O_9_.

Our studies of phase formation during the sintering of SnO_2_ with high additions of cobalt oxide and niobium oxide (much above the solid solubility limit of Co and Nb in SnO_2_) have shown that two secondary phases form in the system; the columbite-type CoNb_2_O_6_ and Co_4_Nb_2_O_9_. While CoNb_2_O_6_ is only a transient phase that reacts with SnO_2_ above 1200°C, the results of EBSD and TEM analyses revealed that larger grains of the Co_4_Nb_2_O_9_ phase remain stable up to 1300°C. SnO_2_ grains start to coarsen above the Co_4_Nb_2_O_9_ formation temperature and frequently develop epitaxial OR with the Co_4_Nb_2_O_9_ grains. Although these phases were not detected in the low-doped ceramics (Tominc *et al.*, 2018 ▸), it may be anticipated that they do form also in the low-doped samples in the regions of local chemical inhomogeneities during the heating and act as seeds for oriented growth of SnO_2_. In the following, we explain the formation of contact and cyclic twins in the dual-doped SnO_2_-based ceramics based on the structural relationship between SnO_2_, CoNb_2_O_6_, and Co_4_Nb_2_O_9_. Reasons for a fairly high fraction of untwinned grains in the low-doped ceramics are also discussed.

### 3.3. Twinning in SnO_2_ in the presence of CoNb_2_O_6_ and Co_4_Nb_2_O_9_

#### 3.3.1. Structural relationship between SnO_2_, CoNb_2_O_6,_ and Co_4_Nb_2_O_9_

**Figure 5:**
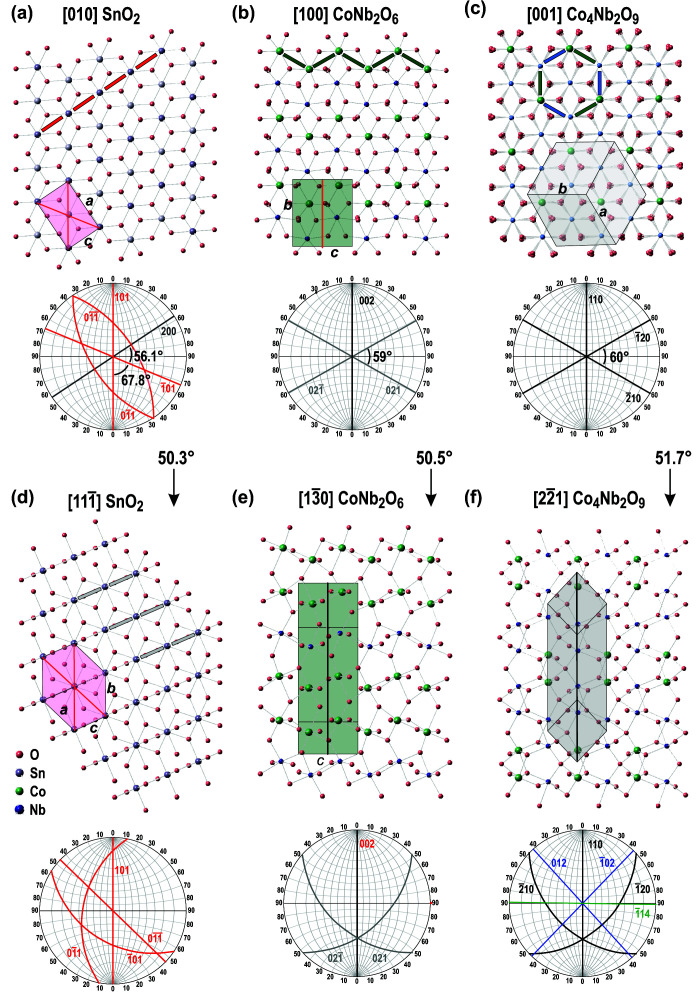
Structures of (*a*,*d*) SnO_2_, (*b*,*e*) columbite-type CoNb_2_O_6_ and (*c*,*f*) Co_4_Nb_2_O_9_ phase with structure coparable to that of corundum in orientations relevant for understanding the formation of (*a*)–(*c*) contact and (*d*)–(*f*) alternating cyclic twins in cassiterite by oriented growth of SnO_2_ on the two structurally related phases. Typical pattern (projected unit cell) of each phase is drawn on the structural models and these patterns will be used for schematic illustration of epitaxial twinning in Figs. 6 ▸–8 ▸ ▸. A stereogram with the most relevant lattice planes involved in the twinning process is shown below each structural model.

Both secondary phases, CoNb_2_O_6,_ and Co_4_Nb_2_O_9_, that form during sintering of dual-doped SnO_2_ are key to constraining the twinning relations in cassiterite. Fig. 5 ▸ shows crystal structures of the three phases oriented along the zone axes that are most important for understanding the formation of contact and subsequent coplanar and alternating cyclic twins in cassiterite. The relevant structural data for the three phases are given in Table 2 ▸.

Cassiterite is structurally related to CoNb_2_O_6_ and Co_4_Nb_2_O_9_ through the hexagonal close-packed (hcp) oxygen substructure. The three phases have different arrangements of the cations within the close-packed layers [Figs. 5 ▸(*a*)–5 ▸(*c*)]. In cassiterite [Fig. 5 ▸(*a*)], the close-packed oxygen layers are severely corrugated and extend in two directions normal to each other, *i.e.* along the *a* and *b* axes [the (200)_Cst_ and (020)_Cst_ planes] (Baur, 1981 ▸). Hence, the sixfold symmetry axis along the direction of the close-packed layers is lost and a new fourfold axis that extends along the *c*-axis of the structure is created (West & Bruce, 1982 ▸). The loss of the sixfold axis can be observed as a deviation of the angles between the three arrays of oxygen atoms [the (002)_Cst_, (101)_Cst,_ and 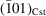 planes in cassiterite] within a close-packed layer from the ideal 60° [see stereogram in Fig. 5 ▸(*a*)]. In a close-packed layer of cassiterite, one-half of octahedral sites are occupied by Sn^4+^ cations, and these form infinite chains of edge-shared octahedra along the *c*-axis.

In columbite-type CoNb_2_O_6_ [Fig. 5 ▸(*b*)] the close-packed oxygen layers lie along the *a* axis, *i.e*. along the (600)_Col-t_ planes. CoNb_2_O_6_ is structurally similar to scrutinyite (α-PbO_2_), a high-temperature polymorph of PbO_2_ (Weitzel, 1976 ▸). Within a single close-packed layer, the cations form a zigzag pattern of edge-shared octahedra. Co^2+^ and Nb^5+^ ions occupy subsequent layers in the 1:2 ratio; two layers are occupied by Co^2+^ and every third layer by Nb^5+^. This causes tripling of the unit cell along the *a* axis. Octahedra in the neighboring close-packed layers are corner-shared. The distortion of oxygen arrays along the close-packed layers is smaller than in cassiterite, the angle between the (021)_Col-t_ and 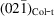 edge-on planes is around 59°. Co_4_Nb_2_O_9_ has trigonal structure comparable to that of corundum, in which two-thirds of the available octahedral interstices are occupied within the hcp oxygen substructure. Typical of the corundum-type structure is arrangement of the cations in a honeycomb pattern within a close-packed layer (Lee & Lagerlof, 1985 ▸). In Co_4_Nb_2_O_9_, the Co^2+^ and Nb^5+^ occupy the octahedral interstices in 2:1 ratio on average [Fig. 5 ▸(*c*)]: two out of three subsequent close-packed layers have mixed composition with Co:Nb in 1:1 ratio, whereas every third layer is occupied only with Co cations, which yields overall Co:Nb ratio of 2:1. Here, oxygen atoms within the close-packed layer are symmetrically arranged and the equivalent (110)_Crn-t_ attice planes intersect exactly at 60°.

Orientation of the SnO_2_ and Co_4_Nb_2_O_9_ in schemes of Figs. 5 ▸(*a*) and 5 ▸(*c*) is based on the results of EBSD and TEM analyses (Fig. 4 ▸) where it was confirmed that (101)_Cst_ planes are parallel to (110)_Crn-t_ planes. This OR is central to understanding the formation of cyclic twins in cassiterite. Another possible OR would be with the *c* axis of cassiterite parallel to the {110}_Crn-t_ planes of the corundum-type phase: [010]_Cst_ || [001]_Crn-t_ and (200)_Cst_ || (110)_Crn-t_; however, no evidence for the existence of this R was found in our samples and also the high lattice mismatch between these two planes (Table 2 ▸) is not in favor to the formation of this OR. Orientation of the columbite-type CoNb_2_O_6_ phase [Fig. 5 ▸(*b*)] with (101)_Rt_[010]_Rt_ || (002)_Col-t_[100]_Col-t_ was selected based on the OR reported by Wittkamper *et al.* (2017 ▸).

The [010]_Cst_ orientation [Fig. 5 ▸(*a*)] is important for understanding the formation of coplanar cyclic twins of cassiterite, where all twin domains are oriented along a common [010]_Cst_ zone axis, whereas the 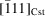 zone axis [Fig. 5 ▸(*d*)] is relevant for studying alternating cyclic twins, where all twin domains have the [111]_Cst_ axis in common. The two orientations are related by rotation along any of the {101}_Cst_ planes for 50.3°. In cassiterite viewed along the [101]_Cst_ zone axis, one pair of {101}_Cst_ planes is edge-on oriented, these planes intersect at 67.8° [see stereogram in Fig. 5 ▸(*a*)] and limit the number of subsequent twin boundaries in coplanar cyclic twins of cassiterite to five. Along the [111]_Cst_ zone axis, another pair of {101}_Cst_ planes is edge-on oriented, these planes intersect at 46.5° [see stereogram in Fig. 5 ▸(*d*)] and limit the number of subsequent twin boundaries in alternating cyclic twins of cassiterite to seven (Padrón-Navarta *et al.*, 2020 ▸). In [111]_Cst_ zone axis, the unit-cell projection of cassiterite may be drawn by a squashed hexagon with {101} planes as the longer diagonals. To study the role of CoNb_2_O_6_ and Co_4_Nb_2_O_9_ in the formation of alternating twins, both structures are shown in orientations obtained by equivalent rotation as SnO_2_. Rotation of CoNb_2_O_6_ along the (002)_Col-t_ planes yields CoNb_2_O_6_ along the 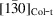 [Fig. 5 ▸(*e*)] whereas, after 51.7° rotation of Co_4_Nb_2_O_9_ along the [110]_Crn-t_ direction, the structure is oriented along the 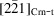 zone axis.

#### 3.3.2. Twinning in SnO_2_ and its relation to the columbite-type CoNb_2_O_6_

Columbite-type CoNb_2_O_6_ is structure-similar to α-PbO_2_, which is also the structural type of high *p-T* modification of rutile, TiO_2_. It has been shown that {101} twins of rutile, found as inclusions in garnets contain epitaxial α-PbO_2_ modification of TiO_2_ at the twin contact with the following OR: [100]_α-TiO2_ || [100]_Rt_; (001)_α-TiO2_ || (011)_Rt_ (Hwang *et al.*, 2000 ▸; Meng *et al.*, 2008 ▸). Wittkamper *et al.* (2017 ▸) studied the growth of SnO_2_ on polished polycrystalline columbite-type CoNb_2_O_6_ substrate with random grain orientation by pulsed laser deposition at 700°C. They found that SnO_2_ develops in two polymorphic modifications on substrate CoNb_2_O_6_ grains, as rutile-type or as α-PbO_2_-type SnO_2_ modification, depending on the orientation of the substrate CoNb_2_O_6_ grains. In grains that promoted the growth of rutile-type SnO_2_, the following OR was determined between SnO_2_ and CoNb_2_O_6_ (c*, columbite indexed by nonstandard *Pcnb* space group): (101)_Cst_[010]_Cst_ || (010)_c*_ [001]_c*_ The OR is identical to the OR between α-PbO_2_-type TiO_2_ and rutile TiO_2_ in {101} twins of rutile in garnets (Hwang *et al.*, 2000 ▸; Meng *et al.*, 2008 ▸).

**Figure 6:**
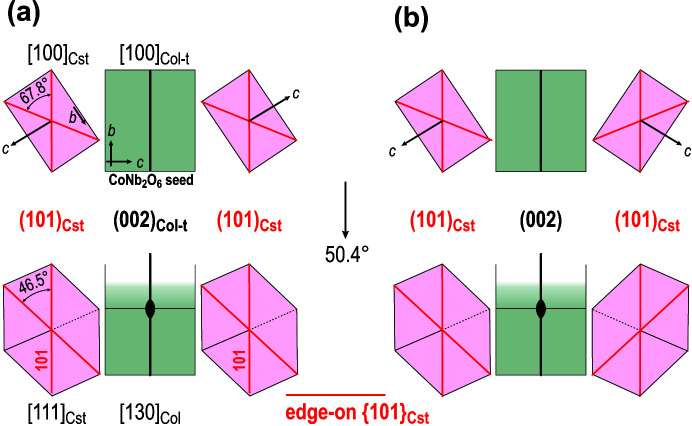
Oriented growth of SnO_2_ (purple) on CoNb_2_O_6_ (greenish) seed can result in (*a*) identical orientation of SnO_2_ on opposite sides of the CoNb_2_O_6_ seed particle leading to the formation of untwinned cassiterite or (*b*) in mirror orientation for the formation of contact (101) twin.

Both findings support our assumption that columbite-type CoNb_2_O_6_ particles can act as seed grains for epitaxial growth of SnO_2_ in the dual-doped ceramics. CoNb_2_O_6_ is the first secondary phase that forms between cobalt and niobium oxides during the firing of doped SnO_2_-based ceramics already around 700°C [see Fig. 3 ▸(*a*)]. Wittkamper *et al.* (2017 ▸) have shown that SnO_2_ grows epitaxially on columbite-type CoNb_2_O_6_ already at temperatures as low as 700°C. A similar process of epitaxial nucleation of SnO_2_ on CoNb_2_O_6_ seed particles may occur in polycrystalline ceramics, where heterostructural nucleation of SnO_2_ on CoNb_2_O_6_ seed in identical or in mirror orientation may be anticipated (Fig. 6 ▸). Nucleation in identical orientation results in the formation of normal (untwinned) cassiterite grains [Fig. 6 ▸(*a*)], whereas nucleation in mirror orientation results in the formation of a contact {101} twin [Fig. 6 ▸(*b*)]. Such composite heterostructural particles that are established at lower sintering temperatures represent nuclei for the development of SnO_2_ contact twins during accelerated grain growth above 1300°C [see densification curve for the dual-doped ceramics in Fig. 1 ▸ (red curve) of Tominc *et al.* (2018) ▸]. An additional effect that occurs in the high-temperature sintering stage is the reaction between CoNb_2_O_6_ and SnO_2_. This causes dissolution of columbite seed grains into the cassiterite matrix grains and hence, the presence of secondary phase grains at the TBs or in the form of overgrown inclusions inside cassiterite grains is never observed. Our previous HR-STEM analyses of TBs and regions in the vicinity of the contacts have revealed non-uniform local enrichment with Co and Nb and also inhomogeneous distribution of both elements inside the SnO_2_ matrix (Tominc *et al.*, 2018 ▸). This supports the hypothesis about epitaxial growth of SnO_2_ on preexisting nanosized CoNb_2_O_6_ seeds and their later recrystallization and diffusion of Co^2+^ and Nb^5+^ into the cassiterite matrix.

#### 3.3.3. Twinning in SnO_2_ and its relation to the Co_4_Nb_2_O_9_

**Figure 7:**
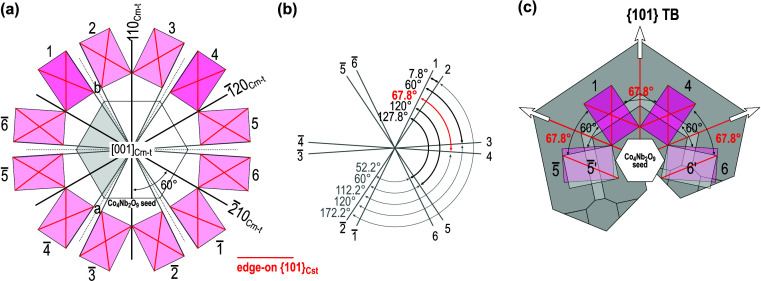
(*a*) Twelve possible (six unique) orientations of cassiterite (purple) in [010]_Cst_ orientation on Co_4_Nb_2_O_9_ seed (gray) in [001]_Crn-t_ orientation. (*b*) Angles between *c* axes of the domains, at 67.6° angle, the domains are in (101) twin orientation. (*c*) Formation of domains 1 and 4 gives a contact twin. The formation of additional SnO_2_ domains for a coplanar twin on the same seed grain requires epitaxial growth of SnO_2_ at 60° (domains 6 and 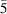 ) and local recrystallization of these domains to 6′ and 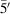 orientation to yield the (101) TB contacts.

In addition to the CoNb_2_O_6_ particles with the columbite-type structure, also the Co_4_Nb_2_O_6_ phase grains with structure comparable to that of corundum can act as seed grains for the nucleation of cassiterite twins. The results of EBSD and TEM analyses have revealed that the following OR preferentially occurs between SnO_2_ and Co_4_Nb_2_O_9_: (101)[010]_Cst_ || (110)[001]_Crn-t_ (Fig. 4 ▸). The small mismatch between (101)_Cst_ and (110)_Crn-t_ planes (Table 2 ▸) is advantageous for the development of this OR. Epitaxial growth of SnO_2_ on Co_4_Nb_2_O_9_ seed grains in this OR can follow 12 possible orientations as schematically shown in Fig. 7 ▸(*a*). Theoretically, any two SnO_2_ domains that grow on a single crystal seed particle may form a (symmetric) interface. This results in 144 combinations, and the contacts can be classified into four types: (i) low-angle tilt boundaries at 7.8° and complementary angles of 172.2° (contacts 1–2 and analogous); (ii) non-crystallographic contacts at 60° and 120° (1–3, 1–5 and analogous), (iii) {101} TBs at 67.8° (contacts 1–4 and analogous) and (iv) {301} TBs at 127.8° (contacts 1–6 and analogous). Also, nucleation of cassiterite in identical orientation resulting in the formation of single-crystal cassiterite at 0° or 180° (the structure is centrosymmetric) may occur (1–1, 2–2…). The possibilities are schematically presented in Fig. 7 ▸(*b*); a more detailed description of this process is given in the description of rutile exsolutions from matrix ilmenite, FeTiO_3_ [see Table 1 ▸ and the corresponding explanation given by Rečnik *et al.* (2015 ▸)].

Epitaxial growth of SnO_2_ domains in various orientations on a Co_4_Nb_2_O_9_ seed grain can result in the formation of different non-crystallographic and crystallographic contacts between SnO_2_ domains. However, the only contact between cassiterite domains that we found in the dual-doped ceramics are crystallographic (101) TBs. This implies that this interface is the most energetically stable and develops/grows further after epitaxial growth of SnO_2_ domains in twin orientation on the seed particle. We even assume that neighboring domains that form at angles close to the (101) TBs (*e.g.* 60°) may recrystallize to twin contacts at the beginning of grain growth. The energy of (101) and (301) twin boundaries with different local stacking was calculated for rutile, TiO_2_ by Lee *et al.* (1993 ▸) and they found that reflection twin with ½〈111〉 in-plane translation is the lowest energy interface in rutile. Since TiO_2_ and SnO_2_ are isostructural, we may assume that (101) TBs are low-energy interfaces also in cassiterite. The prevalence of (101) TBs in our ceramics samples and also the abundance of (101) twins over (301) twins in natural cassiterite crystals are also in favor of this hypothesis. Epitaxial growth of cassiterite in {101} twin orientation on Co_4_Nb_2_O_9_ seed can result in the formation of simple contact twins, *i.e.* between cassiterite domains 1–4, 2–5 or 3–6 in Fig. 7 ▸(*a*). Each of these simple contact twins may represent the basis for the development of a multiple cyclic twin with either coplanar or alternating configuration.

Let us now focus on the formation of coplanar multiple cyclic twins. In our samples, we observed grains with three or four domains although theoretically coplanar cyclic twins with six domains separated by five subsequent {101} TBs and an additional non-crystallographic contact to close the cyclic twin are possible (Padrón-Navarta *et al.*, 2020 ▸). Nucleation of a cyclic twin cannot be explained by epitaxial growth of SnO_2_ domains in identical OR on a Co_4_Nb_2_O_9_ seed. We believe that the formation of a coplanar cyclic twin starts with the formation of a contact twin, for example between domains 1 and 4 [Fig. 7 ▸(*c*)]. The formation of additional domains on a single-crystal Co_4_Nb_2_O_9_ seed grain in twin orientation with the domains 1 and 4 and, at the same time, in the expected OR between SnO_2_ and Co_4_Nb_2_O_9_ (with {101}_Cst_ parallel to {110}_Crn-t_) is not possible. The closest possibility is epitaxial growth of SnO_2_ domains on Co_4_Nb_2_O_9_ at 60° angles on both sides of the starting contact twin (domain 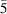 next to 1 and or domain 6 next to 4). These would initially form energetically demanding non-crystallographic contacts at 60°, which would then locally recrystallize to (101) twin contact (domain 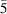 → 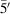 and 6 → 6′). Such occurrence on only one side of the 1–4 contact twin would lead to the formation of a coplanar twin with three domains, whereas the occurrence of the process on both sides of the starting 1–4 contact twin would lead to the formation of a coplanar twin with four domains separated by three TB contacts [*e.g.* domains 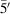 –1–4–6′, Fig. 7 ▸(*c*)]. In any case, each cyclic twin is closed by the formation of a short NCC between the end domains [*e.g*. 5′ and 6′ in Fig. 7 ▸(*c*)].

**Figure 8:**
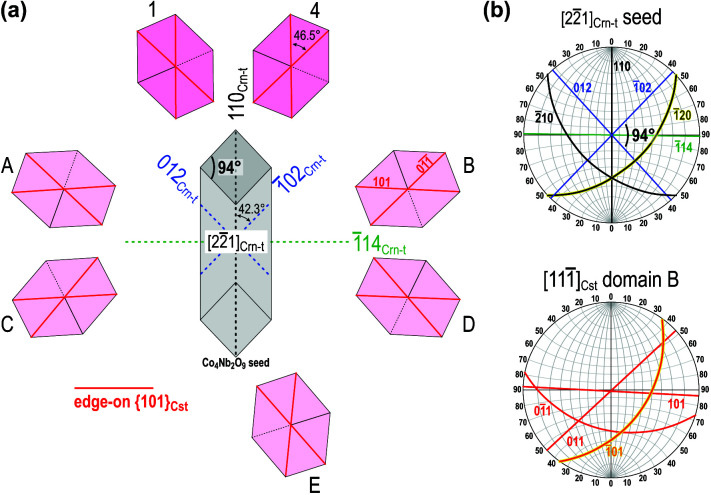
(*a*) Scheme of an alternating twin where all cassiterite domains are oriented along their [111]_Cst_ zone axis and the Co_4_Nb_2_O_9_ seed is oriented along the pseudo fourfold 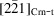 zone axis. (*b*) Stereograms of the Co_4_Nb_2_O_9_ seed and SnO_2_ domain B show that besides the edge-on 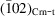 and 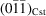 planes, another pair of planes 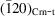 and 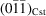 lies oblique to the viewing direction (marked by yellow). This pair of planes corresponds to the expected SnO_2_–Co_4_Nb_2_O_9_ OR.

Besides coplanar cyclic twins, a comparable fraction of alternating cyclic twins always forms in the ceramics (Padrón-Navarta *et al.*, 2020 ▸) indicating that the probability for the formation of coplanar and alternating twins is similar. We may presume that the initial structural element, *i.e.* a contact {101} twin on a Co_4_Nb_2_O_9_ particle can also develop into an alternating multiple cyclic twin, where all twin domains have the [111]_Cst_ as the common zone axis. In order to understand the crystallographic relationship between SnO_2_ domains and the Co_4_Nb_2_O_9_ seed, it is beneficial to study the phases in rotated view (Fig. 8 ▸). Fig. 8 ▸(*a*) is a scheme of alternating cassiterite twin with the maximum possible number of twin domains (*i.e.* seven) which are separated by six twin boundary contacts. In the dual-doped ceramics, twins with three or four subsequent domains are the most common, whereas twins with five or more domains are quite rare (Padrón-Navarta *et al.*, 2020 ▸). This suggests that conditions for the formation of a fully developed alternating twin of cassiterite in sintered ceramics are hardly ever achieved.

Cassiterite domains in Fig. 8 ▸(*a*) are oriented along their [111]_Cst_ zone axes, whereas the seed is in the 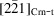 orientation. Cassiterite domains 1 and 4 and the Co_4_Nb_2_O_9_ seed represent the basic element for the development of twins with coplanar [Fig. 7 ▸(*a*)] or alternating morphology. Here, the OR between cassiterite domains 1 and 4 and the seed can alternatively be written as 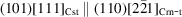 (see also Figs. 4 ▸, 5 ▸(*d*) and 5 ▸(*f*)]. A common characteristic of all other cassiterite domains (marked by letters A-E) is that they have one of the oblique (101)_Cst_ lattice planes nearly parallel to one of the oblique (110)_Crn-t_ planes of the Co_4_Nb_2_O_9_ seed, which corresponds to the expected SnO_2_–Co_4_Nb_2_O_9_ OR; an example for SnO_2_ domain B and the Co_4_Nb_2_O_9_ seed is shown on the stereograms in Fig. 8 ▸(*b*). This OR between cassiterite and corundum may lead to the stabilization of (101) TBs between cassiterite domain B and domain 1 as the low-energy interface and subsequent recrystallization to a cyclic twin with alternating morphology. It is also interesting that in this orientation, the corundum-type structure exhibits pseudo-cubic symmetry with (110)_Crn-t_ planes (*d* = 0.2587 nm) nearly perpendicular to 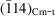 planes (*d* = 0.2776 nm), whcih coincides with the pseudo eightfold symmetry of the alternating twin and may additionally contribute to the stabilization of cyclic twins with alternating morphology.

### 3.4. Microstructure development and the formation of different types of twins in SnO_2_-based ceramics co-doped with cobalt and niobium oxides

The main goal of this work was to understand the formation of contact and multiple cyclic twins of cassiterite which commonly form when in SnO_2_-based ceramics when SnO_2_ is sintered with small additions of CoO and Nb_2_O_5_ (1 mol% of each dopant; Co:Nb = 1:2). Exactly this composition, in terms of the total amount of the dopants and the Co:Nb ratio, yields microstructure with the highest density, composed of large SnO_2_ grains with a high fraction of twins (Tominc *et al.*, 2018 ▸). The microstructure contains about two thirds of untwinned grains and the rest are cassiterite twins, most of which are contact (101) twins, while about 6% of all grains are multiple cyclic twins with coplanar or alternating morphologies that occur in comparable fractions (Padrón-Navarta *et al.*, 2020 ▸). The fact that abundant twinning and especially the formation of multiple cyclic twins is not observed in the samples without the addition of both dopants implies that twinning in the dual-doped cassiterite is related to a combination of specific chemical and thermodynamic conditions during the high-temperature sintering of SnO_2_ in the presence of both oxides.

It has been observed in several oxides and alloys that the addition of specific dopants/ impurities triggers the formation of crystallographic contacts like twin boundaries and similar interfaces during grain/crystal growth. For example, the formation of growth twins on {111} planes is observed in silicon flakes during their growth in aluminium–silicon eutectic alloy as a result of the addition of sodium and other modifiers (Lu & Hellawell, 1987 ▸); the formation of basal-plane inversion boundaries (IBs) in ZnO is triggered by the addition of Sb_2_O_3_, SnO_2_, and other oxides (Rečnik *et al.*, 2001*a* ▸; Daneu *et al.*, 2000 ▸; Schmid *et al.*, 2013 ▸), abundant formation of {111} twins in MgAl_2_O_4_ spinel is observed during crystal growth in the presence of BeO (Drev *et al.*, 2013 ▸) antiphase boundaries typically form in non-stoichiometric or doped oxide perovskites (*e.g.* Rečnik *et al.*, 2001*b* ▸). Characteristic of these impurity-induced planar defects is enrichment of the interface with the dopant atoms and these atoms reside in specific interstitial positions within the boundary. The local atomic structure of these interfaces is well ordered and contains structural elements found in the secondary phase that forms between the main phase and the dopant, *i.e.* spinel phases in the case of IBs in ZnO, taaffeite modulated phase in the case of twins in BeO-doped MgAl_2_O_4_ spinel and polytypic Ruddlesden–Popper phases in the case of antiphase boundaries in perovskites. The formation of impurity-induced planar defects is an energetically favorable process and can be described as continuous adsorption of impurity atoms to the growth interface which results in the progressive formation of a 2D monolayer with different chemical compositions inside the host crystal (Lu & Hellawell, 1987 ▸). The process has a profound effect on grain/crystal growth and the development of ceramic microstructures. Grains in which the impurity-induced interfaces nucleate at the beginning of crystal growth exhibit exaggerated growth, their growth is significantly faster in the direction of the defect and these grains may also develop anisotropic morphologies, at least in the initial stages of crystal growth.

If the twin boundaries in co-doped cassiterite that we studied in this work would be classic impurity-induced defects, we would expect to find Co and/or Nb ordered inside the twin contact planes. However, the results of our previous study (Tominc *et al.*, 2018 ▸) have shown that the twin boundaries in co-doped cassiterite contain only irregular segregation of Co and Nb to the vicinity of the twin boundaries, while an ordered arrangement of the dopants within the interface was not observed. Also, these twin boundaries do not show exaggerated growth to such extent as observed in the systems with the impurity-induced defects and this suggests that these defects form via a different mechanism. An important clue for understanding the formation of twins in co-doped cassiterite ceramics is the crystallographic relationship between SnO_2_ and the Co_4_Nb_2_O_9_ secondary phase: (101)[010]_Cst_ || (110)[001]_Crn-t_ that was found in the sample with higher dopant additions (Fig. 4 ▸). In natural rutile twins, the twin boundaries sometimes contain up to few nanometres thick layers or inclusion of structurally related phases that indicate the formation of these twins is by epitaxial growth on structurally related phases or by oriented (topotaxial) recrystallization of a structurally related precursor mineral. For example, a thin epitaxial layer of ilmenite was found at (301) rutile twin contacts (Daneu *et al.*, 2007 ▸), whereas discrete oriented corundum-inclusions were found at (101) twin boundaries (Daneu *et al.*, 2014 ▸), both in hydro­thermally formed rutile crystals. On the other hand, (101) twins of rutile found as inclusions in high *P-T* garnets contain a nanometre-thick slab of α-PbO_2_-type TiO_2_ at the interface (Hwang *et al.*, 2000 ▸; Meng *et al.*, 2008 ▸). Structural characteristics of epitaxial secondary phases found at the twin contacts are important indicators of geochemical conditions during the rutile crystal growth/formation.

**Figure 9:**
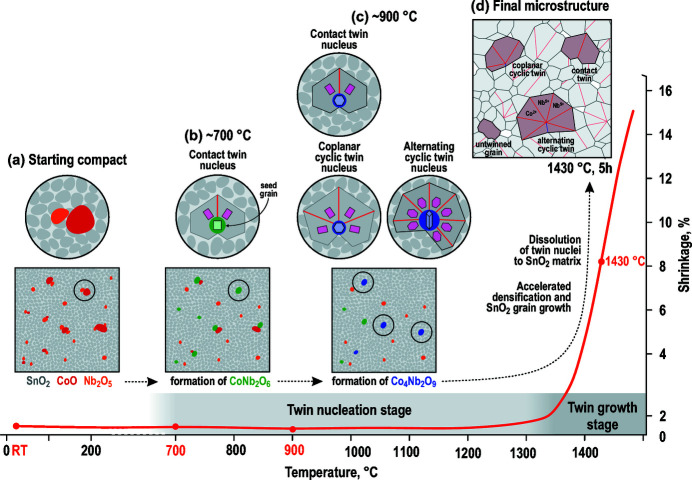
The formation of different types of twins in dual-doped SnO_2_ during heating up to 1430°C; red line is the sample densification curve according to Tominc *et al.* (2018 ▸). (*a*) Inhomogeneous distribution of the dopants (CoO, red; Nb_2_O_5_, orange) among nanosized SnO_2_ (gray) particles the starting compact. (*b*) The formation of CoNb_2_O_6_ seed particles (green) and contact twins nuclei at around 700°C and (*c*) the formation of Co_4_Nb_2_O_9_ seeds (purple) and contact and multiple cyclic twins nuclei at around 900°C. (*b*) and (*c*) represent the twin nucleation stage, which is bound to limited densification at temperatures up to ∼1300°C. (*d*) The twin growth stage coincides with accelerated densification and SnO_2_ grain growth above 1300°C. During this stage, the twin nuclei develop into cassiterite twins due to the fast growth of (101) twin contacts. The seed grains recrystallize to SnO_2_, Co^2+^ and Nb^5+^ are incorporated into SnO_2_ grains in the form of solid solution and seed grains are no longer present at the TBs and in the cyclic twin cores in the form of crystalline particles.

The results of our study suggest that twins in co-doped cassiterite ceramics form according to a similar mechanism that involves oriented epitaxial growth of SnO_2_ domains on grains of secondary CoNb_2_O_6_ and Co_4_Nb_2_O_9_ phases. These particles represent heterogeneities in the system, which act as seeds and lower the energy barrier for epitaxial growth of SnO_2_ (Lee *et al.*, 2016 ▸). The structural similarities between SnO_2_ and both secondary phases favor the formation of heterointerfaces. The presence of seed grains is overwritten during the high-temperature sintering when solid-state diffusion processes that govern coarsening of cassiterite grains (twin growth stage) become activated. The only (indirect) evidence for the presence of seed grains that trigger the nucleation of twins in the initial stages of crystal growth is irregular segregation of Co and Nb to the twin boundaries (Tominc *et al.*, 2018 ▸) and local enrichment of the cyclic twin cores as shown in this work [Fig. 2 ▸(*c*)]. The process is presented in Fig. 9 ▸ and described in more detail in the continuation.

The starting pellet contains mostly nanosized SnO_2_ powder homogenized with the addition of 1 mol% of CoO and 1 mol% of Nb_2_O_5_ (Co:Nb ratio of 1:2). The distribution of both dopants in the starting pellet is not perfectly homogenous and areas with locally higher concentrations of cobalt oxide (with larger particles in the starting powder) are likely [Fig. 9 ▸(*a*)]. The first reaction that occurs during the heating is the formation of the CoNb_2_O_6_ phase at around 700°C. Due to the close structural relationship between CoNb_2_O_6_ and SnO_2_, the SnO_2_ grain in the vicinity of the CoNb_2_O_6_ phase grains can locally recrystallize onto these grains in the orientation for (101) twins [Fig. 9 ▸(*b*)]. The formation of Co_4_Nb_2_O_9_ phase starts at around 900°C in areas locally enriched with cobalt oxide with the reaction between CoNb_2_O_6_ and excess cobalt oxide. These particles represent seeds for the formation of contact and multiple cyclic twins [Fig. 9 ▸(*c*)]. In the temperature range up to 1300°C, densification of the sample is very limited, the sample behaves similarly to undoped SnO_2_, where sintering in the low-temperature range (500–1000°C) is controlled by surface diffusion, and shrinking is not observed (Leite *et al.*, 2001 ▸). The only processes observed in this temperature range are reactions between cobalt and niobium oxides to CoNb_2_O_6_ and Co_4_Nb_2_O_9_ secondary phases and these reactions do not have an influence on the densification. The secondary phase particles, however, represent seeds for local recrystallization of SnO_2_, which results in the formation of twin nuclei for the grwoth of different types of twins at higher sintering temperatures. The genesis of twins in the nucleation stage as a result of the presence of impurities has been suggested already by Senechal (1980 ▸).

At temperatures above 1300°C, densification of the sample is strongly enhanced [Fig. 9 ▸(*d*)]. It is related to accelerated solid-state diffusion due to the formation of point defects as a result of aliovalent doping as described by Tominc *et al.* (2018 ▸). During these processes, Co and Nb are incorporated into the SnO_2_ matrix in the form of solid solution. The total addition of CoO and Nb_2_O_5_ in the low-doped sample (1 mol% of each dopant) is around or below the solid solubility limit of the dopants in SnO_2_ matrix (our estimated value of total Co and Nb solid solubility in SnO_2_ is ∼6 at%), therefore almost all dopants are incorporated into the SnO_2_ in the low-doped sample. The columbite-type CoNb_2_O_6_ phase grains will in any case react with the SnO_2_ to the trirutile phase above 1200°C, therefore these grains, which represent the nuclei for contact twins readily dissolve into SnO_2_. This explains the local enrichment in Co and Nb around the twin boundaries as observed in our previous work (Tominc *et al.*, 2018 ▸). Dissolution of the Co_4_Nb_2_O_9_ grains into the SnO_2_ matrix phase along with the formation of secondary Co_2_SnO_4_ spinel phase particles in the reaction between excess cobalt oxide and SnO_2_ can also occur in the low-doped samples, where the total dopant addition does not exceed the solid solubility limit. In our previous study we found some residual niobium oxide and Co_2_SnO_4_ spinel phase particles at the grain boundaries and triple points. Both can be attributed to imperfect homogenization of the starting powders and limited diffusion during solid-state sintering. The development of cyclic twins with eccentrically positioned nuclei with long TBs and a short NCC between the first and the last twin domain is a result of the fast growth of low-energy (101) TB contacts and very limited growth of the energetically unfavorable NCC.

The described sequence of chemical reactions and epitaxial growth processes not only offers a plausible explanation for the formation of contact and multiple cyclic twins by the ‘epitaxially induced twinning’ mechanism during the sintering of dual-doped SnO_2_-based ceramics, but also explains the presence of untwinned grains, which represent two-thirds of all grains. Untwinned grains may simply coarsen without an epitaxial contact to a secondary cobalt–niobium oxide grain, by epitaxial growth of a single SnO_2_ domain on a seed, by the growth of two or more SnO_2_ domains in identical or inconvenient orientation for the formation of a (101) twin contact on a seed particle. It is also important to emphasize that higher dopant additions do not enhance the formation of twins because we found many untwinned grains also in the sample with much higher addition of both dopants [see Fig. 4 ▸(*a*)]. Even more interesting, this sample contained fewer multiple cyclic twins in comparison to the low-doped sample, and twins with maximum four twin domains were observed. This suggests that small, nanosized nuclei are necessary for the formation of multiple cyclic twins.

## 4. Conclusions

The results of this study indicate that the formation of contact and multiple cyclic twins in SnO_2_-based ceramics co-doped with cobalt and niobium oxides is not accidental but triggered by oriented growth of SnO_2_ domains in twin orientations on structurally related nanosized seed particles of secondary phases via the so-called ‘epitaxially-induced twinning’ mechanism.

The formation of twins is a two-stage process. The twin nuclei form in the twin nucleation stage below 1300°C, where only local recrystallization of SnO_2_ on CoNb_2_O_6_ and Co_4_Nb_2_O_9_ seed particles occurs. These particles have crystal structures closely related to SnO_2_ and enhance the formation of heteroepitaxial contacts with SnO_2_ domains in different orientations. In the twin nucleation stage, some of the SnO_2_ domains are in (101) twin orientation and contacts between these domains develop into (101) TBs. These TBs then grow preferentially in the twin growth stage above 1300°C, when densification and SnO_2_ grain growth are strongly accelerated as a result of diffusion processes related to the incorporation of Co and Nb into the SnO_2_ matrix. This stage involves recrystallization/dissolution of the seed grains into the matrix SnO_2_ and obliterates the evidence about the twin nucleation stage.

One of the most important implications of this study is that the absence of a twin nucleus in a growth twin does not necessarily imply that it was not present in the twin nucleation stage. Detailed analyses of the twin contacts and twin cores, especially in the case of multiple cyclic twins provide valuable evidence about the presence of seed particles in the nucleation stage of the twin formation.

## Supplementary Material

XRD patterns of 50% SnO2 + 50% (Co3O4 + 3Nb2O5) and 50% SnO2 + 50% (4Co3O4 + 3Nb2O5) compositions after sintering at different temperatures. DOI: 10.1107/S2052520622006758/yh5020sup1.pdf

## Figures and Tables

**Table:** (*a*) Compositions for the formation of the columbite-type CoNb_2_O_6_ phase X (SnO_2_) + (100-X) (1Co_3_O_4_ + 3Nb_2_O_5_); Co:Nb = 1:2; *X* in mol%.

SnO_2_		Co_3_O_4_		Nb_2_O_5_	
*X* (mol%)	wt%	mol%	wt%	mol%	wt%
90	83.937	2.50	3.725	7.50	12.338
75	63.527	6.25	8.459	18.75	28.014
50	36.733	12.50	14.673	37.50	48.594

**Table:** (*b*) Compositions for the formation of the corundum-type Co_4_Nb_2_O_9_ phase Y(SnO_2_) + (100 − *Y*)(4Co_3_O_4_ + 3Nb_2_O_5_); Co:Nb = 2:1;Y in mol%.

SnO_2_		Co_3_O_4_		Nb_2_O_5_	
*Y* (mol%)	wt%	mol%	wt%	mol%	wt%
90	84.357	5.714	8.557	4.286	7.086
75	64.254	14.286	19.556	10.714	16.190
50	37.470	28.571	34.207	21.429	28.323

**Table 2:** Structural data for SnO_2_, CoNb_2_O_6_ and Co_4_Nb_2_O_9_ relevant for understanding the formation of contact and cyclic twins in cassiterite

Phase	Basic structural data	Relevant lattice planes and spacings	Epitaxial relationships and lattice mismatches
SnO_2_ (Cassiterite)	ICSD 84576		
	Tetragonal; *P*42/*mnm*	*d*(101)_Cst_ = 2.644 Å	** [010]_Cst_ || [100]_Col-t_**
	*a* = 4.737 Å	*d*(200)_Cst_ = 2.398 Å	(101)_Cst_ || (002)_Col-t_; 4.7%
	*c* = 3.185 Å		(101)_Cst_ || (021)_Col-t_; 4.8%
CoNb_2_O_6_	ICSD 15854		(200)_Cst_ || (021)_Col-t_; 6.2%
	Orthorhombic; *Pbcn*	*d*(002) = 2.522 Å	---------------------------------
	*a* = 14.148 Å	*d*(021) = 2.485 Å	
	*b* = 5.712 Å		
	*c* = 5.045 Å		** [010]_Cst_ || [100]_Crn-t_**
Co_4_Nb_2_O_9_	ICSD 27134		(101)_Cst_ || (110)_Crn-t_; 2.1%
	Trigonal, 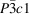	*d*(110) = 2.589 Å	(200)_Cst_ || (110)_Crn-t_; 8.9%
	*a* = 5.177 Å	*d*(102) = 3.788 Å	101)_Cst_ || (104)_Crn-t_; 4.9%
	*c* = 14.168 Å	*d*(104) = 2.776 Å	

## Footnotes

1 Abbreviation for columbite is not in the list of Whitney & Evans ( 2010 ▸); we decided to use Col.

## References

[bb1] Armbruster, T. (1981). *N. Jahrb. Miner.* **7**, 328–334.

[bb2] Aruga, A., Tokizaki, E., Nakai, I. & Sugitani, Y. (1985). *Acta Cryst.* C**41**, 663–665.

[bb3] Baur, W. H. (1981). *Mater. Res. Bull.* **16**, 339–345.

[bb4] Beck, H. P. (2012). *Z. Kristallogr. Cryst. Mater.* **227**, 843–858.

[bb5] Bertaut, E. F., Corliss, L., Forrat, F., Aleonard, R. & Pauthenet, R. (1961). *J. Phys. Chem. Solids*, **21**, 234–251.

[bb6] Castellanos, R. M. A., Bernès, S. & Vega-González, M. (2006). *Acta Cryst.* E**62**, 117–119.

[bb7] Daneu, N., Rečnik, A., Bernik, S. & Kolar, D. (2000). *J. Am. Ceram. Soc.* **83**, 3165–3171.

[bb8] Daneu, N., Rečnik, A. & Mader, W. (2014). *Am. Mineral.* **99**, 612–624.

[bb9] Daneu, N., Schmid, H., Rečnik, A. & Mader, W. (2007). *Am. Mineral.* **92**, 1789–1799.

[bb10] Drev, S., Rečnik, A. & Daneu, N. (2013). *CrystEngComm*, **15**, 2640.

[bb11] Force, E. R., Richards, R. P., Scott, K. M., Valentine, P. C. & Fishman, N. S. (1996). *Can. Mineral.* **34**, 605–614.

[bb12] Goldschmidt, H. J. (1960). *Metallurgia*, **62**, 211–218.

[bb13] Grimmer, H. (1979). *Scr. Metall.* **13**, 161–164.

[bb14] Hahn, Th. & Klapper, H. (2006). *International Tables for Crystallography*, Vol. D, *Physical properties of crystals*, edited by A. Authier, ch. 3.3, pp. 393–448. Dordrecht: Kluwer.

[bb15] Hielscher, R. & Schaeben, H. (2008). *J. Appl. Cryst.* **41**, 1024–1037.

[bb16] Hwang, S. L., Shen, P., Chu, H. T. & Yui, T. F. (2000). *Science*, **288**, 321–324. 10.1126/science.288.5464.321 10764642

[bb17] Kawamura, F., Kamei, M., Yasui, I. & Sunagawa, I. (1999). *J. Am. Ceram. Soc.* **82**, 774–776.

[bb18] Krakow, R., Bennett, R. J., Johnstone, D. N., Vukmanovic, Z., Solano-Alvarez, W., Lainé, S. J., Einsle, J. F., Midgley, P. A., Rae, C. M. F. & Hielscher, R. (2017). *Proc. R. Soc. A*, **473**, 20170274. 10.1098/rspa.2017.0274 PMC5666230 29118660

[bb19] Lee, J., Yang, J., Kwon, S. G. & Hyeon, T. (2016). *Nat. Rev. Mater.* **1**, 16034.

[bb20] Lee, W. E. & Lagerlof, P. D. (1985). *J. Elec. Microsc. Tech.* **2**, 247–258.

[bb21] Lee, W. Y., Bristowe, P. D., Gao, Y. & Merkle, K. L. (1993). *Philos. Mag. Lett.* **68**, 309–314.

[bb22] Leite, E. R., Cerri, J. A., Longo, E., Varela, J. A. & Paskocima, C. A. (2001). *J. Eur. Ceram. Soc.* **21**, 669–675.

[bb23] Lu, S. & Hellawell, A. (1987). *Metall. Trans. A*, **18**, 1721–1733.

[bb24] Mainprice, D., Bachmann, F., Hielscher, R. & Schaeben, H. (2015). *Geol. Soc. London Spec. Publ.* **409**, 251–271.

[bb25] Meng, D. W., Wu, X. L., Sun, F., Huang, L. W., Liu, F., Han, Y. J., Zheng, J. P., Meng, X. & Mason, R. (2008). *Micron*, **39**, 280–286. 10.1016/j.micron.2007.07.001 17698363

[bb26] Morawiec, A. (1997). *Acta Cryst.* A**53**, 273–285.

[bb27] Navrotsky, A., Ma, C., Lilova, K. & Birkner, N. (2010). *Science*, **330**, 199–201. 10.1126/science.1195875 20929770

[bb28] Otálora, F. & García-Ruiz, J. M. (2014). *Chem. Soc. Rev.* **43**, 2013–2026. 10.1039/c3cs60320b 24264125

[bb29] Padrón-Navarta, J. A., Barou, F. & Daneu, N. (2020). *Acta Cryst.* B**76**, 875–883. 10.1107/S2052520620010264 PMC7535064 33017320

[bb30] Pan, X. Q. & Fu, L. (2001). *J. Appl. Phys.* **89**, 6048–6055.

[bb31] Penn, R. L. & Banfield, J. F. (1998). *Science*, **281**, 969–971. 10.1126/science.281.5379.969 9703506

[bb32] Rečnik, A., Čeh, M. & Kolar, D. (2001*b*). *J. Eur. Ceram. Soc.* **21**, 2117–2121.

[bb33] Rečnik, A., Daneu, N., Walther, T. & Mader, W. (2001*a*). *J. Am. Ceram. Soc.* **84**, 2657–2668.

[bb34] Rečnik, A., Stanković, N. & Daneu, N. (2015). *Contrib. Mineral. Petrol.* **169**, 19.

[bb35] Schmid, H., Okunishi, E. & Mader, W. (2013). *Ultramicroscopy*, **127**, 76–84. 10.1016/j.ultramic.2012.07.014 22898248

[bb36] Senechal, M. (1980). *Sov. Phys. Crystallogr.* **25**, 520–524.

[bb37] Shahani, A. J. & Voorhees, P. W. (2016). *J. Mater. Res.* **31**, 2936–2947.

[bb38] Stanković, N., Rečnik, A. & Daneu, N. (2016). *J. Mater. Sci.* **51**, 958–968.

[bb39] Suzuki, K., Ichihara, M. & Takeuchi, S. (1991). *Philos. Mag.* **63**, 657–665.

[bb40] Tominc, S., Rečnik, A., Samardžija, Z., Dražić, G., Podlogar, M., Bernik, S. & Daneu, N. (2018). *Ceram. Int.* **44**, 1603–1613.

[bb41] Weitzel, H. (1976). *Z. Kristallogr.* **144**, 238–258.

[bb42] West, A. R. & Bruce, P. G. (1982). *Acta Cryst.* B**38**, 1891–1896.

[bb43] Whitney, D. L. & Evans, B. W. (2010). *Am. Mineral.* **95**, 185–187.

[bb44] Wittkamper, J., Xu, Z., Kombaiah, B., Ram, F., De Graef, M., Kitchin, J. R., Rohrer, G. S. & Salvador, P. A. (2017). *Cryst. Growth Des.* **17**, 3929–3939.

[bb45] Zheng, G., Pan, X. Q., Schweizer, M., Zhou, F., Weimar, U., Göpel, W. & Rühle, M. (1996). *J. Appl. Phys.* **79**, 7688–7694.

