# Supplementary material for: Formation of contact and multiple cyclic cassiterite twins in SnO2-based ceramics co-doped with cobalt and niobium oxides
Source: Acta Crystallogr B Struct Sci Cryst Eng Mater. 2022 Jul 27;78(Pt 4):695–709. doi: 10.1107/S2052520622006758 (PMC9370213; doi:10.1107/S2052520622006758)

STRUCTURAL SCIENCE
CRYSTAL ENGINEERING
MATERIALS

Volume 78 (2022)

Supporting information for article:

Formation of contact and multiple cyclic cassiterite twins in SnO2-based ceramics co-doped with cobalt and niobium oxides

Nina Daneu, Goran Dražič, Matjaž Mazaj, Fabrice Barou and José Alberto Padrón-Navarta

Supporting information

S1: XRD patterns of the (a) 50% SnO_2 + 50% (Co_3O_4 + $3Nb_2O_5$) and (b) 50% SnO_2 + 50% ($4Co_3O_4$ + $3Nb_2O_5$) compositions after sintering at different temperatures.

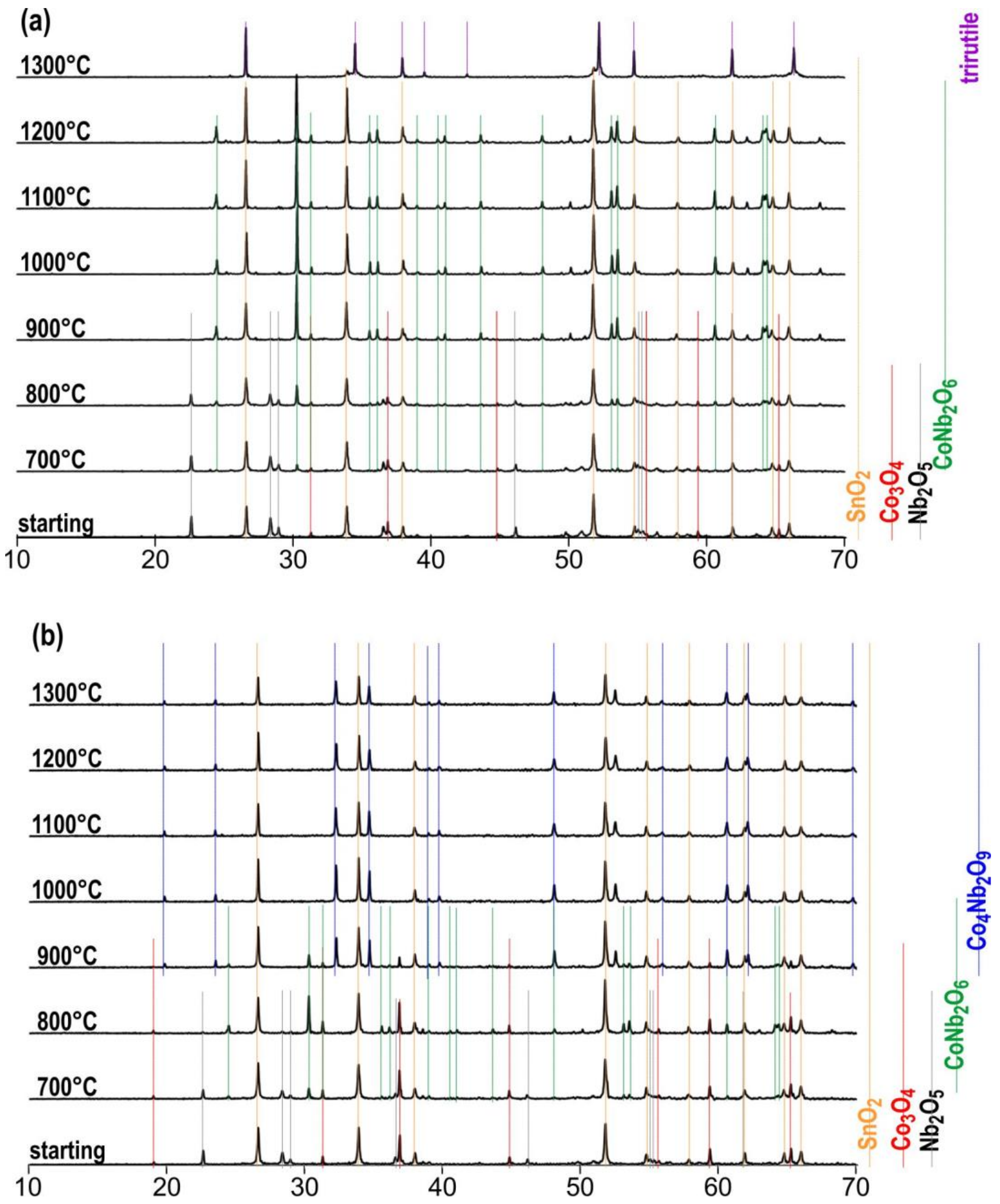

Supplement: Supplementary file 1 [file b-78-00695-sup1.pdf]
